# Worsening of Verbal Fluency After Deep Brain Stimulation in Parkinson's Disease: A Focused Review

**DOI:** 10.1016/j.csbj.2016.11.003

**Published:** 2016-11-27

**Authors:** Andreas Højlund, Mikkel V. Petersen, Kousik Sarathy Sridharan, Karen Østergaard

**Affiliations:** Center of Functionally Integrative Neuroscience (CFIN), Dept. of Clinical Medicine, Aarhus University, Denmark; Dept. of Neurology, Aarhus University Hospital, Denmark

**Keywords:** Deep brain stimulation, Parkinson's disease, Verbal fluency

## Abstract

Worsening of verbal fluency after treatment with deep brain stimulation in Parkinson's disease patients is one of the most often reported cognitive adverse effect. The underlying mechanisms of this decline are not well understood. The present focused review assesses the evidence for the reliability of the often-reported decline of verbal fluency, as well as the evidence for the suggested mechanisms including disease progression, reduced medication levels, electrode positions, and stimulation effect vs. surgical effects. Finally, we highlight the need for more systematic investigations of the large degree of heterogeneity in the prevalence of verbal fluency worsening after DBS, as well as provide suggestions for future research.

## 1 Introduction

This focused review was invited as a result of the II. International Conference on Deep Brain Stimulation (Düsseldorf, March 2016), and it aims to provide an up-to-date status on the incidence and potential explanations for the often-reported verbal fluency (VF) decline after deep brain stimulation (DBS) in Parkinson's disease (PD), as well as a set of pointers for future research. Several explanations have been proposed including disease progression, reduced medication levels, microlesions, as well as electrode location and stimulation itself, but with no clear conclusions drawn so far. Advancing our understanding of this aspect of DBS contributes to the continued improvement of the DBS treatment, as well as to our understanding of the effect mechanisms behind DBS.

The timeliness of this focused review has allowed us to include three recently published meta-analyses on neuropsychological adverse effects (including VF worsening) after DBS in PD [12], [80], [81]. As revealed by Combs et al. [12], there are relatively few studies assessing VF declines after DBS in the internal globus pallidus (GPi) compared to DBS in subthalamic nucleus (STN), which is also mirrored in this review. This underrepresentation of GPi studies is reflective of a general tendency in the field to prefer STN to GPi as target for DBS in PD [63], as well as of potential differences in cognitive adverse effects between the two targets [12].

The structure of this review centers around two overarching questions:

1. What is the evidence for verbal fluency (VF) worsening after DBS-treatment in PD?
2. What are the possible mechanisms underlying such a decline?

In response to 1, we will review the evidence for the commonly reported VF decline in relation to pre- and post-surgery evaluations for both STN- and GPi-DBS, as well as highlight the large degree of heterogeneity in the incidence of VF worsening following DBS, which has not been investigated systematically yet.

In response to 2, we will review the literature in relation to suggested explanations such as disease progression, reduced medication levels, electrode positions, and stimulation vs. lesion effects.

## 2 Background

PD is a progressive neurodegenerative disease characterized by the motor symptoms rest tremor, postural instability, rigidity and bradykinesia (slowness of movement) and a variety of non-motor symptoms including cognitive decline and worsening of VF [53], [86].

DBS in STN and GPi has been shown to effectively alleviate PD patients' motor symptoms when medication is no longer a viable treatment [17], [21], [32], [36], [46], [87], [88]. However, the effects of DBS on cognition are still not well understood [79]. And as already mentioned, one of the most consistently reported detrimental effects of DBS in PD is a worsening of VF [12], [48], [69], [79], [80], [81]. VF deficits are also part of the PD symptomatology prior to DBS surgery [24], but the underlying cause of the worsening after DBS is still an open question.

Verbal fluency is tested with a task requesting the patient, within a minute, to name as many words as possible starting with a specific letter (e.g., F, A, or S; known as phonemic or letter fluency) or stemming from a given category (e.g., animals; known as semantic or category fluency) [8], [35]. Deficits in verbal fluency may thus come about from both linguistic and executive dysfunctions as it involves a multitude of cognitive processes including lexical search, memory retrieval, executive functioning, and response monitoring, inhibition, and selection [35], [59].

## 3 Evidence for Worsening of Verbal Fluency After DBS

When assessing the evidence for VF worsening after DBS, it is important to note the point raised by Woods et al. [78] that far from all studies reporting on cognitive sequelae of DBS include the sufficient sample sizes to detect even large effect sizes. In fact, in their sample of 30 published studies between 1997 and 2004, only two studies did. This urges caution in interpreting the results of most individual studies on this topic and places a strong emphasis on the results of carefully conducted meta-analyses, and in the absence of such on the results from well-powered randomized control trial (RCT) studies.

Fortunately, in relation to the evidence for VF worsening after DBS, two meta-analyses have aptly summed up the available literature on pre- and post-surgery evaluations of the cognitive sequelae of DBS at least three months after surgery.

Parsons et al. [48] conducted a meta-analysis on 28 studies from 1990 to 2006 on STN-DBS meeting inclusion criteria which included reporting of change scores and neuropsychological evaluations at baseline and follow-up. Among the 28 studies, 16 reported data for phonemic VF (355 patients), and 16 reported data for semantic VF (337 patients), summing up to 21 studies in total reporting on phonemic and/or semantic VF. On the basis of this, they found average effect sizes of moderate size (0.51 and 0.73) for both phonemic and semantic VF declines.

Combs et al. [12] extended Parsons et al.'s [48] meta-analysis from 2006 by analyzing studies with baseline and follow-up neuropsychological evaluations from both STN- and/or GPi-DBS treatments in PD. These meta-analyses revealed that both targets resulted in moderate effect size declines in both phonemic and semantic VF. However, the available evidence for the effects of GPi-DBS on VF are still relatively sparse, and therefore the observed slight disadvantage for STN is inconclusive. In their meta-analyses on STN-DBS and GPi-DBS, there are, however, a few inconsistencies. First, there are overlapping study cohorts (Ardouin et al. [5] and Pillon et al. [50]; as well as Daniels et al. [15] and Witt et al. [74]). Second, the reported total number of studies included vs. those listed in the overview table do not exactly match ([12], Table 1). And third, the total numbers of patients reported for the phonemic VF task for both STN-DBS and GPi-DBS exceed the total sums of included study patients in the overview table ([12], Tables 1–3). Nonetheless, these inconsistencies are minor, and we deem the reported results credible.

There is thus reliable evidence for a worsening of moderate effect size in both phonemic and semantic VF after STN-DBS. The evidence for a similar decline in GPi-DBS is still too sparse to be considered reliable, but there are subtle tendencies suggesting a slight disadvantage for STN (when considering other cognitive adverse effects, as well).

Following the publication of the results from the large RCT study on STN- and GPi-DBS by the CSP-468 Study Group ([21], [55], [70], [71], the debate on which target – STN or GPi – to select for DBS in PD has received renewed attention [42], [73].

## 4 Suggested Causes of Worsening of Verbal Fluency

### 4.1 Disease Progression

In order to assess the continued disease progression as a potential explanation of the reported VF declines, studies are needed which include a matched PD control group on best medical treatment (BMT) with VF testing at similar baseline and follow-up intervals as the DBS group. Very recently, two meta-analyses were conducted on such studies comparing VF declines in STN-DBS PD patients and in PD patients on BMT [80], [81]. Both meta-analyses seem to confirm that PD patients after STN-DBS treatment experience VF worsening to a larger extent (i.e., moderate to small effect sizes) than matched PD patients on BMT. However, these results should be interpreted with considerable caution due to substantial methodological issues in both meta-analyses.

First, Wyman-Chick [80] included eligible studies published between 2000 and June 2014, but only 9 out of 140 identified studies met the study's inclusion criteria for phonemic VF and also only 9 for semantic VF (i.e., in total, 10 studies were included: 8 with both phonemic and semantic, 1 with only phonemic, and 1 with only semantic VF data). Furthermore, the author relied on comparisons of the two groups' VF scores only at the follow-up evaluation (and not the groups' change scores). But a difference in follow-up scores is not necessarily reflective of a difference in change scores. Both Marshall et al. [38] and Zangaglia et al. [85] are examples of this discrepancy. In Marshall et al. [38] neither phonemic nor semantic VF changes were significantly different between the DBS-treated and BMT groups (*p* = 0.41 and *p* = 0.60, respectively). However, when only the follow-up values were included in Wyman-Chick's [80] meta-analysis, the differences between the two groups were assigned adjusted effects sizes of −0.33 and −0.21 for phonemic and semantic VF, respectively, denoting small, but substantial, differences between the two groups at follow-up. Zangaglia et al. [85] reported a significant difference in phonemic VF scores between the two groups at the 36-month-follow-up. However, there was already a noticeable difference between the two groups at the baseline, albeit non-significant, and the STN-DBS PD group's phonemic VF scores did not change significantly between baseline and follow-up (*p* = 0.164). Hence, none of the included differences in follow-up VF scores from the two studies adequately reflect a reduction in VF scores due to the DBS treatment compared to BMT.

Second, Xie et al. [81] included studies published until June 2015 and focused on potential differences in the two groups' change scores. For the VF deficits, this meant that only 6 and 4 out of 172 identified articles were included for phonemic and semantic VF, respectively (these numbers are available in the article's supplementary material). Unfortunately, the authors included both Witt et al. [75] and Daniels et al. [15] as separate studies, yet these are overlapping cohorts (Witt et al. [75] analyzed a subset of the patients in Daniels et al. [15]). Furthermore, it seems the authors selected the wrong standard deviation (SD) values from the study by Castelli et al. [9] and Rothlind et al. [55]. They wrongfully interpreted the SD values of the mean values at the follow-up evaluations as belonging directly to the change scores. Castelli et al. [9] is also included in Wyman-Chick's [80] meta-analysis where she has interpreted exactly the same SD values as belonging to the mean values at the follow-up evaluation. Furthermore, it is not clear why only the phonemic (and not also semantic) VF values were included from Cilia et al. [11], Merola et al. [40], and Rothlind et al. [55] (where semantic VF values are listed under “Processing speed” in Table 3), and vice-versa for the semantic (but not phonemic) VF values from Williams et al. [72], when both sets of VF values were readily available in all four studies. Including these values could have increased the number of properly included studies for both VF scores to six (when also accounting for the overlap between Witt et al. [75] and Daniels et al. [15]).

Hence, both meta-analyses suffer from relatively low power ([81], in particular), as well as from substantial methodological issues. We therefore consider their combined evidence relatively inconclusive.

However, if we focus on the two RCT studies included in the meta-analyses, i.e., Witt et al. [74] and Rothlind et al. [55], they both provide evidence in the form of well-powered direct comparisons of the change scores of both DBS and BMT groups. Both report significant worsening of both phonemic and semantic VF in the DBS groups compared to the BMT group between baseline and after 6 months. In fact, Rothlind et al. [55] included both an STN- and a GPi-DBS group, and both groups showed very similar declines in VF after DBS compared to the BMT group. Hence, disease progression does not seem to be able to account for the observed worsening of VF after DBS, regardless of target.

### 4.2 Reduced Dopaminergic Medication Levels

STN-DBS (but not GPi-DBS) is often followed by a significant reduction in dopaminergic medication [21]. Based on this general observation as well as a correlation between greater reduction in dopaminergic levels and greater worsening of phonemic VF in their own study, Sáez-Cea et al. [56] suggested that reduced medication levels may play a role in the observed VF declines. However, to the best of our knowledge, only one study [22] has reported that PD patients OFF dopaminergic medication performed worse on (semantic) VF than healthy controls whereas there was no significant difference between the two groups when the patients were ON medication. This could suggest a beneficial role of dopaminergic medication on VF performance (as briefly mentioned by Cools [13] with reference to Gotham et al. [22]), and by extension a detrimental role of reduced medication levels in the observed VF decline after STN-DBS. But Gotham et al. [22] also reported no significant difference within the PD group on (semantic) VF performance for ON and OFF dopaminergic medication, which, in essence, is the crucial and most sensitive contrast in this respect, and thus not suggestive of an effect of dopaminergic medication on VF performance.

Nonetheless, since changes in dopaminergic medication levels between baseline and follow-up are often compared to the observed declines in VF after DBS, this allowed the aforementioned meta-analyses by Parsons et al. [48] and Combs et al. [12] to also test for such a relation. Neither of them found any relation between reductions in medication levels and VF decline following DBS. And even though this is in essence a null result, the combined evidence of the two meta-analyses strongly suggests that reduced levels of dopaminergic medication (after STN-DBS) cannot account for the observed VF declines after DBS.

### 4.3 Electrode Positions

A few studies have investigated the effects of electrode locations on the observed worsening of VF after DBS. And even though the evidence is still sparse, this factor seems to affect the VF performance after DBS to a larger degree than disease progression and reduced medication levels.

Witt et al. [75] observed a significant worsening of semantic VF in a group of STN-DBS PD patients compared to a PD control group on BMT. By dividing the STN-DBS group into decliners and stable performers, they found that the active contacts of 75% (9 out of 12) of the decliners lay outside the pseudo-volume created on the basis of the active contacts of the 19 stable performers. Especially in the left hemisphere, most of the decliners' active contacts were also placed more ventrally.

Okun et al. [46], on the other hand, altered the active contact for stimulation in both unilateral STN-DBS and GPi-DBS patients in order to test the effects of a more dorsal contact, a more ventral contact, the optimal contact and OFF stimulation (i.e., four settings in total). They observed no effects of this manipulation on VF, but they did observe a decline between baseline and follow-up in phonemic VF in the STN group across all four settings (which was greater than the GPi group, but the contrast did not reach their predefined *p* < 0.025 level of significance). On the basis of observing the non-significant worsening of VF also in the OFF stimulation condition, the authors suggested an insertion effect rather than stimulation per se as the cause of this decline. However, based on a subset of the STN-DBS patients from the very same cohort, Okun's group [41] subsequently reported on correlations between volume of tissue activated (VTA) and phonemic VF decline. Here, stimulation of larger ventral parts of STN was correlated with worse VF performance [41]. And in a further follow-up study on the GPi-DBS patients, Okun's group [19] showed that stimulation region did not affect VF performance in a subset of the GPi-DBS patients [19], who also did not show any significant declines in VF after DBS.

Furthermore, the patients included in the COMPARE trial and reported by Okun et al. [46], as well as by Mikos et al. [41] and Dietz et al. [19], were all unilaterally implanted with either STN-DBS or GPi-DBS. Hence, testing stimulation of different contact positions with bilateral stimulation could potentially have greater effect on VF performance than those reported by Okun et al. [46].

Ehlen et al. (2014) found that STN-DBS PD patients' changes in VF performance between ON and OFF stimulation correlated with electrode location and stimulation amplitude. Better VF performance in ON than OFF was associated with more antero-medial positions and higher stimulation amplitudes, which suggests at least some active component in the stimulation itself. We note, however, that this suggested effect of the stimulation itself was beneficial to VF performance, rather than detrimental. And since the study did not include any baseline measurements of the patients' VF performance before surgery, it is difficult to know how these beneficial effects of stimulation were related to any potential worsening of VF performances compared to pre-surgery baseline.

Finally, York et al. [83] also found correlations between VF declines and electrode locations of variable kinds. More superior and lateral locations in the left hemisphere seemed to be associated with greater phonemic VF declines. In the right hemisphere, greater phonemic VF declines were associated with electrodes located more posterior and superior, but laterally closer to STN. And greater semantic VF declines were correlated with more superior locations in the right hemisphere. These results are not straightforward to interpret as they rely on a multitude of correlations with a relatively small sample size, but they still suggest associations between electrode locations and the observed VF declines.

The available evidence on effects of electrode locations on the observed worsening of VF after DBS is still preliminary and inconclusive. But when detailed VTA-modeling is taken into account as in Mikos et al. [41], or decliners are compared to stable performers in a volumetric space as in Witt et al. [75], electrode positions do seem to play a role in VF decline following DBS – in STN, at least.

### 4.4 Stimulation vs. Surgery

Even though the evidence is not overwhelming, the correlations between electrode locations and the observed VF declines suggest that either the stimulation itself or insertion effects from the surgery may affect VF performance after DBS in STN. Unfortunately, the sparse literature on this matter is also inconclusive, but it does seem to suggest that both the stimulation and the surgery itself may have effects on the observed worsening of VF after DBS.

Wojtecki et al. [77] showed that the frequency of stimulation of STN had opposite effects on motor symptoms and verbal fluency in PD patients. Low frequency stimulation at 10 Hz improved VF performance while worsening the motor symptoms compared to the typical high frequency stimulation at 130 Hz, which improved motor symptoms while worsening VF performance. This suggests an active role of the stimulation frequency, and by extension the stimulation itself, in the VF performance of STN-DBS-treated PD patients.

However, in a more recent open label RCT study, Okun et al. [45] employed a study design with a delayed DBS activation group as control group. 25% of the implanted patients were randomly assigned to a control group where the DBS would not be turned on until 3 months after surgery. Interestingly, the authors found that both groups showed worsening of phonemic and semantic VF after 3 months, a worsening that was sustained after 12 months in both groups. This evidence, on the other hand, strongly suggests an effect of surgery, rather than stimulation.

When it comes to testing ON and OFF stimulation effects on VF performance, one study has shown significant differences in VF performance between ON and OFF stimulation with worse performance during ON [57], supporting the notion of an active role of the stimulation. In contrast to this, as already mentioned Okun et al. [46] did not observe any significant differences between ON and OFF stimulation, despite a general (but non-significant) VF decline with STN-DBS after surgery, perhaps suggestive of an effect of insertion from surgery rather than of the actual stimulation. And yet the few other studies that have tested ON and OFF stimulation in relation to VF show mixed results between phonemic and semantic VF but with incomplete reporting (e.g., lack of baseline, use of test composite scores, lack of tests on the relevant contrasts) due to which we cannot fully assess the similarity of the observed VF declines or lack thereof during ON and OFF stimulation [20], [28], [43], [50].

Smith et al. [61] addressed the potential effects of microlesions by using the number of micro-eletrode (MER) passes during surgery as an index of the extent of the microlesion in STN from the surgery, and they did not find any significant correlations between the number of MER passes and the phonemic VF decline after DBS.

Common to the few studies reporting no difference in VF performance during ON and OFF stimulation – and hence suggesting insertion effects – is that their evidence is based on negative results. But such null results do not provide very conclusive evidence since the absence of evidence is not evidence of absence. Equivalence testing [54], [58] or Bayesian statistics [18], [33], on the other hand, provide statistical frameworks that allow the researcher to interpret such null results in a more systematic and meaningful manner.

Furthermore, most of the studies testing ON and OFF stimulation effects only allowed 10–30 min before starting neuropsychological testing after turning OFF or changing the stimulation [46], [50], [77], or do not report how long they waited [20], [28], [43], [57]. This is a relatively short interval considering that the cardinal PD motor symptoms vary between a few minutes and several hours in how quickly they are alleviated/reappear after turning ON/OFF the DBS [65]. With a similar design studying response inhibition, Hershey et al. [26] observed differences between unilateral activation of a more dorsal and more ventral contact during a Go/NoGo-task after waiting at least 42 min between change of stimulation settings and testing. Hence, when employing the ON vs. OFF stimulation design, or when testing the effect of stimulation in different active contacts, it may be advisable to wait at least 45 min [26], and perhaps even 2 h considering the motor symptoms [65], before testing VF or other neuropsychological measures.

Thus, it is still unclear from the literature to what extent the observed VF declines after DBS are caused by insertion effects from the surgery or caused by the actual stimulation itself. But nonetheless, stimulation and insertion effects in combination with electrode locations are those of the suggested mechanisms behind the VF decline that show the strongest associations with the observed worsening of VF after DBS.

### 4.5 Patient Inherent Risks for VF Worsening

This focused review deals with the reported VF worsening after DBS, i.e., in the PD patient cohorts that are screened and found eligible for DBS and who then receive the treatment. This means that it does not deal with the potentially increased risks for VF worsening (and other cognitive declines) in PD patients that are deemed too old or too cognitively impaired to receive DBS.

Results from two RCT studies [15], [60] have suggested that advanced age, low levodopa response and higher levodopa equivalent dose (LED) at baseline were associated with cognitive decline after DBS. However, as noted by Daniels et al. [15], their three factors (higher age, higher LED and higher axial subscore on UPDRS-III at baseline) only explained about 23% of the variance in the cognitive decline after DBS. Furthermore, both studies made use of composite scores for their measures of cognitive decline, and their results are therefore not directly transferrable to the reported VF worsening after DBS, which is of focus in this review.

And importantly, both the aforementioned meta-analyses of VF worsening after DBS by Parsons et al. [48] and Combs et al. [12] reported that none of the investigated risk factors were related to VF worsening after DBS. Parsons et al. [48] tested age, disease duration, stimulation parameters, and LED change after surgery as moderators of the VF decline. Combs et al. [12] tested age, disease duration, LED at baseline, and UPDRS score off medication at baseline in relation to the reported VF worsening. Hence, it does not seem that any of the potential patient inherent risks in the DBS-treated PD cohorts can account for the observed VF worsening after surgery.

## 5 Heterogeneity in Prevalence

As already alluded to, there is considerable heterogeneity in the prevalence of the worsening of VF after DBS. It seems that a subset of patients (10–40%) are often driving the reported group effects of VF decline [7], [14], [31].

Unfortunately, far from all studies report proper assessments of this individual variation, e.g., reliable change indices (RCIs; [27], [67]), but the studies that do include RCIs for pre- and post-surgery evaluations all report a small but substantial subgroup of patients with reliable declines, whereas the rest of the DBS patients experience no reliable difference in VF or maybe even a slight improvement. Williams et al. [72] reported that 26% and 29% of STN-DBS PD patients showed reliable declines in phonemic and semantic VF, respectively. The same numbers for their GPi-DBS group were 11% and 29%, respectively. Witt et al. [75] reported that 23% and 39% of STN-DBS PD showed reliable declines in phonemic and semantic VF. Rothlind et al. [55] reported that, across both groups of STN- and GPi-DBS, 16.5% and 11% showed reliable declines in phonemic and semantic VF. And they observed no differences in prevalence between the two groups. York et al. [82] reported that 26.1% and 40% of STN-DBS PD showed reliable declines in phonemic and semantic VF. Finally, Zahodne et al. [84] also referred to an observation of heterogeneity in VF declines following unilateral DBS.

To the best of our knowledge, this relatively large degree of individual variation has not received any thorough and systematic attention. And yet it seems that what is consistently reported as a group effect, is mainly driven by a small, but substantial, subgroup of the DBS-treated patients. In our view, this heterogeneity in prevalence seems to hold promising explanatory potential for the worsening of VF after DBS if properly characterized and investigated.

## 6 Possible Underlying Mechanisms

As previously mentioned, VF involves several cognitive processes related to linguistic and executive functioning, in particular [24], [25], [35], [59]. By the use of interference tasks, neurocognitive models have focused on contrasting phonemic and semantic VF performance in an attempt to ascribe them to frontal lobe (executive functioning) and temporal lobe (lexical search) processes, respectively [39], [44].

Lesion studies have refined this proposed dissociation between phonemic and semantic VF. In a meta-analysis on VF performance after focal cortical lesions, Henry & Crawford [25] showed that frontal lesions affected phonemic and semantic VF to similar extents, whereas temporal lobe lesions affected semantic VF more than phonemic VF, suggestive of a shared frontal lobe component in both phonemic and semantic VF. Furthermore, Chouiter et al. [10] recently investigated VF performance in 191 patients with traumatic brain injury (TBI) and managed to also include patients with brain lesions in subcortical structures. This allowed them to show that basal ganglia structures, including putamen, caudate nucleus, and globus pallidus, were integral to both phonemic and semantic VF, which is in line with the reported effects of DBS in STN (and GPi) on VF in PD patients.

To add to this, Troyer et al. [66] suggested on the basis of their study of patients with focal brain lesions that the contributions of frontal lobe and temporal lobe processes were related to switching and clustering, respectively, both of which are subprocesses of VF and not specific to phonemic or semantic VF. Recently, Vonberg et al. [68] analyzed clusters and switches during VF performance with DBS ON and OFF. Here, they showed more switches (and marginally shorter switch times) during DBS ON compared to DBS OFF, but with no significant differences in the total number of words between ON and OFF. The authors interpret these results to suggest that STN-DBS may subtly increase cognitive flexibility in PD patients. However, due to no baseline evaluations it is difficult to fully assess the role of the increased number of switches in relation to potential worsening of VF after DBS. Further supporting our observation of considerable heterogeneity in the prevalence of VF worsening, the authors' inclusion of data on the individual patients' VF performances in the supplementary material confirmed substantial individual differences in the degree to which patients performed better or worse during DBS ON or OFF.

Very tentatively, the limited evidence from the literature seems to suggest that STN (and GPi) may be involved in VF performance through a basal-ganglia-thalamocortical network [29], [64] involving mainly dorsolateral prefrontal cortex (dlPFC, BA 9, 46) and left inferior frontal gyrus (l-IFG, BA 44–45) at the cortical level as is suggested by the few available PET studies on VF in DBS-treated PD patients [31], [57]. This subthalamo-frontocortical connection is further supported by a recently published study by Wojtecki et al. [76] combining recordings of local field potentials (LFP) in the STN through externalized DBS electrodes and EEG scalp recordings. Preliminary results from five PD patients demonstrated enhanced coherence between STN and frontal cortex in the low-frequency bands (alpha-theta, 5–15 Hz) during a verbal generation task [76].

## 7 Directions for Future Research

Crucially missing from this present overview is more evidence on the effects of stimulation itself and surgery on the reported VF worsening after DBS, as well as on the effects of electrode locations. These aspects entail comparing VF performance during both ON and OFF stimulation conditions at follow-up compared to baseline, as well as relating the potential worsening to detailed VTA-modeling in the individual patients. A few studies have already employed ON/OFF testing including baseline measurements, but this holds for only one of the RCT studies [46]. The total number of such studies does not warrant a meta-analysis as of yet. Hence further studies implementing this study design are needed. And in this regard, more studies making use of the design introduced by Okun et al. [45] with a delayed DBS activation group would allow for further assessments of the potential chronic effects of stimulation which cannot be assessed with an ON/OFF design with relatively short OFF periods (minutes or a few hours).

Furthermore, only very few studies have tested the effects of stimulation while patients were also OFF medication, which is the most optimal way to directly target an actual stimulation effect. Finally, evidence from such study protocols in terms of ‘no significant differences’ between the two conditions is not sufficient in this regard. Equivalence testing or Bayesian inference should be used to address and interpret such potential null results more meaningfully.

Regarding the heterogeneity of the prevalence of VF declines among DBS-treated PD patients, this has not received sufficient attention, why we recommend this aspect to be taken into account in future studies, especially in combination with more detailed VTA-modeling. In this regard, it may not be sufficient to merely compare stimulation in “dorsal” and “ventral” contacts (as in [46]) in order to account for the potential effects of electrode location and stimulation. Anatomical considerations concerning both cortical projections (the hyperdirect pathway from frontal cortex) and subcortical basal ganglia connections to and from the ventro-medial part of STN (referred to as the ‘associative’ subregion) would be of great value in this context. The traditional view of STN anatomy and function divides it into three separate regions, the motor, associative and limbic regions [37]. However, recent primate studies using anterograde tracers suggests noticeable overlaps between these three subsections [4], [23] in addition to a high degree of variation in the overall size and position of the STN in PD patients [16], [52].

Recent methodological advances in both acquisition and processing of diffusion-weighted MRI (DWI) allow us to non-invasively map the structural networks of the brain with a newfound precision [30], [62]. Such diffusion-based tractography has already been used to examine the tissue and pathways targeted in DBS treatment [6], [49]. These advanced techniques allow for detailed delineation of the connections between the STN (and GPi), cortex and other basal ganglia structures at the individual patient level. Several studies in healthy adults have demonstrated how the STN subsections and overlaps can be delineated using tractography [1], [2], [34], [51]. Implementing state-of-the-art tractography methods, combined with VTA-modeling, may allow detailed exploration of the neural pathways stimulated with DBS in individual patients. Further integrating these methodological advances with measures of behavior and neurophysiology (such as VF performance and M/EEG recordings) provides a clear avenue for advancing our knowledge of the mechanisms of DBS and its potential role in the observed worsening of VF after DBS.

In relation to potentially mapping the neural pathways stimulated with DBS in the individual patient, the few functional neuroimaging studies on VF and DBS in PD using PET [31], [57] have shown correlations between reduced activity in left inferior frontal gyrus (IFG) and (left) dorsolateral prefrontal cortex (dlPFC) and worsening of VF as an effect of STN-DBS. The sparse neuroimaging evidence thus supports a more active role of the stimulation itself in the VF decline where STN-DBS may affect this frontal network through its indirect connections to thalamus via GPi [3], [47], [64], or antidromically via the hyperdirect pathway connecting the prefrontal cortex directly to STN [29]. The observed worsening of VF after GPi-DBS could potentially be attributed to similar network via thalamus, but more studies are still needed in order to assess how reliably VF is negatively affected by DBS in GPi.

## 8 Conclusion

Based on recent and earlier meta-analyses, there is reliable evidence for a worsening of both phonemic and semantic VF after DBS. This primarily pertains to STN-DBS since the number of available studies on the cognitive sequelae of GPi-DBS is still too low for drawing reliable conclusions. The effect sizes of the VF worsening are moderate in size, which seems to be tolerable at the group level, but these tolerable effect sizes may also be reflective of more debilitating effects in a subgroup of PD patients with DBS.

There is no clear impression of the possible underlying mechanisms from the literature, but with evidence from PD control groups on best medical treatment (BMT) in two large-scale RCT studies, disease progression does not seem to be able to account for the worsening of VF in DBS patients. Also, DBS-related reductions in dopaminergic medication (mainly in STN-DBS patients) cannot account for the VF decline.

Hence, it seems that either surgery or stimulation itself or both together in combination with the electrode positions are driving factors. However, the evidence in this relation is inconclusive and sparse. The few studies that include detailed VTA-modeling seem to suggest an active role of the stimulation, at least in STN-DBS. But at the same time, the few studies testing VF performance during ON and OFF stimulation failed to find significant differences between the two conditions, tentatively suggestive of an insertion effect from the surgery, rather than stimulation itself. Hence, more studies are needed before a systematic meta-analysis can be conducted.

Finally, we have highlighted an aspect of the literature that has not received systematic attention to date, namely a large degree of heterogeneity in the incidence of VF declines following DBS (in both STN and GPi). We speculate that individual variation in cortical and subcortical connections to and from STN and/or GPi may contribute to this heterogeneity. Hence, the application of advanced tractography in combination with detailed VTA-modeling may provide new insights into the role of stimulation effects vs. effects of surgery.

Our recommendations for future studies on VF include optimizing study designs to include both ON and OFF stimulation as well as baseline measures, calculating reliable change indices (RCI) for neuropsychological results, and acquiring diffusion-weighted MRI on patients for tractography of cortical and subcortical connections to and from STN/GPi.
